# Genome Sequence of a CHeRI Orbivirus 3 Strain Isolated from a Dead White-Tailed Deer (Odocoileus virginianus) in Florida, USA

**DOI:** 10.1128/MRA.00523-20

**Published:** 2020-06-25

**Authors:** Thais C. S. Rodrigues, John A. Lednicky, Julia C. Loeb, Juan M. Campos Krauer, Samantha M. Wisely, Thomas B. Waltzek, Kuttichantran Subramaniam

**Affiliations:** a Department of Infectious Diseases and Immunology, College of Veterinary Medicine, University of Florida, Gainesville, Florida, USA; b Emerging Pathogens Institute, University of Florida, Gainesville, Florida, USA; c Department of Environmental and Global Health, College of Public Health and Health Professions, University of Florida, Gainesville, Florida, USA; d Department of Large Animal Clinical Sciences, College of Veterinary Medicine, University of Florida, Gainesville, Florida, USA; e Department of Wildlife Ecology and Conservation, University of Florida, Gainesville, Florida, USA; KU Leuven

## Abstract

We report the genome sequence of an orbivirus isolated from a dead farmed white-tailed deer in Florida. The deer was coinfected with epizootic hemorrhagic disease virus type 2. Phylogenetic and genetic analyses supported the virus as the fourth strain of the CHeRI orbivirus 3 species.

## ANNOUNCEMENT

Orbiviruses (family *Reoviridae*, genus *Orbivirus*) possess 10 double-stranded RNA genome segments (1) that encode structural (VP1 to VP7) and nonstructural (NS1 to NS4) viral proteins (2, 3). The outer capsid structural proteins (e.g., VP2 and VP5) typically exhibit the greatest genetic variability and determine the orbivirus serotype, whereas the genes that encode the inner capsid structural proteins (e.g., VP3 and VP7) display less genetic variability and are used for species demarcation (4). Bluetongue virus, epizootic hemorrhagic disease virus (EHDV) (5–8), and other orbiviruses (9–11) have recently been isolated from diseased white-tailed deer in Florida.

In December 2018, spleen and whole blood were collected from a 5-month-old dead farmed white-tailed deer (animal identification no. OV895). All work was approved by the Institutional Animal Care and Use Committee (IACUC) at the University of Florida (IACUC protocol no. 201609390 [initiated 24 May 2016] and 201909390 [initiated 21 March 2019]). RNA extracted from the blood using a QIAamp viral RNA minikit (Qiagen) tested positive for EHDV-2 (11). The spleen tissue was processed for virus isolation attempts in C6/36 and Vero E6 cells as described previously (11). Cytopathic effects (CPE) were observed in C6/36 cells at 8 days postinoculation and then in Vero E6 cells at 11 days postinoculation and were reminiscent of those we described previously for mixed EHDV and Cervidae Health Research Initiative (CHeRI) orbivirus infections (11). The CPE first manifested as the formation of cytoplasmic inclusions concomitant with granulation of the cytoplasm, followed by enlargement of the cells, some with fusiform morphology. Infected cells subsequently detached singly or in clumps from the growing surface.

RNA was extracted from clarified C6/36 cell culture medium using a QIAamp viral RNA minikit (Qiagen) and served as the template for the construction of a cDNA sequencing library using a NEBNext Ultra II RNA library preparation kit (New England Biolabs). The resulting cDNA library was sequenced on an Illumina MiSeq system as described previously (10). A total of 1,756,934 reads with an average read length of 240 bp were obtained and *de novo* assembled in CLC Genomics Workbench using default parameters. BLASTX searches of the resulting contigs recovered the complete coding sequences for all 10 segments of an orbivirus and an EHDV-2 strain (data not shown). The total length of the complete coding sequences of the 10 orbivirus segments was 18,705 bp, with a G+C content of 39.22%.

**FIG 1:**
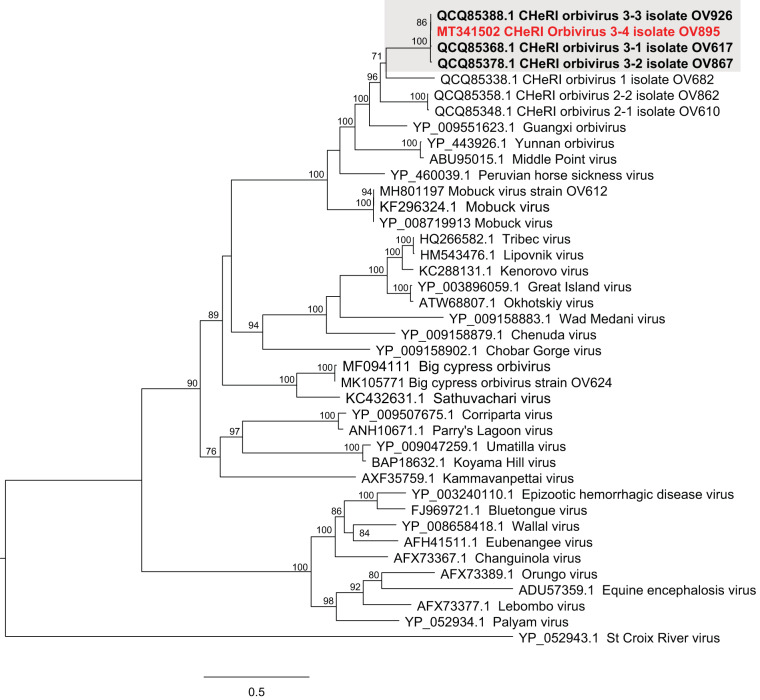
Phylogram depicting the relationship of the CHeRI orbivirus 3-4 (red) to 39 other viruses within the genus *Orbivirus*. The maximum likelihood tree was generated based on the alignment of the amino acid sequences of the innermost subcore capsid protein T2 (VP3), using 1,000 bootstrap replicates. Branch lengths are based on the number of inferred substitutions, as indicated by the scale. The shaded region in the phylogram highlights the CHeRI orbivirus 3 clade. The GenBank accession number and virus name are provided for each orbivirus included in the phylogenetic analysis.

BLASTN analyses of the orbivirus sequences against the NCBI nucleotide database revealed greatest identities with CHeRI orbivirus 3 strains for all segments. The VP1, VP3, VP4, VP6, VP7, NS1, NS2, and NS3 sequence identities ranged from 85.8 to 99.8%. The VP2 and VP5 sequences displayed significantly lower sequence identities (63.6 to 64.7% and 71.7 to 95.6%, respectively) with respect to CHeRI orbivirus 3 strains. An amino acid alignment of the VP3 of the orbivirus with 39 other orbiviruses was performed in Geneious Prime v2019.2.1 and was used to generate a maximum likelihood phylogram in IQ-TREE (http://iqtree.cibiv.univie.ac.at) using default parameters. The orbivirus was supported as a member of the CHeRI orbivirus 3 clade (Fig. 1). Thus, the orbivirus characterized in this study represents a new CHeRI orbivirus 3 strain and likely a new serotype (3).

Here, we report the discovery of a new CHeRI orbivirus 3 strain from a deer coinfected with EHDV-2. Similarly, 4 of 6 deer from which CHeRI orbiviruses were previously isolated were coinfected with EHDV-2 (11). The CHeRI orbiviruses’ closest relatives are mosquito-borne orbiviruses, and CHeRI orbiviruses have been isolated in mosquito cells (11). Thus, CHeRI orbiviruses may represent significant mosquito-borne deer pathogens circulating in farmed populations in Florida. Additional research is needed to understand the pathogenicity of CHeRI orbiviruses with and without EHDV-2 coinfections, as well as the vector(s) involved in transmission.

### Data availability.

The raw sequence data for CHeRI orbivirus 3-4 have been deposited in the NCBI GenBank and Sequence Read Achieve (SRA) databases under accession no. MT341501 to MT341510 and PRJNA629823, respectively.
